# Associations between implementation characteristics and evidence-based practice sustainment: a study of the Adolescent Community Reinforcement Approach

**DOI:** 10.1186/s13012-015-0364-4

**Published:** 2015-12-24

**Authors:** Sarah B. Hunter, Bing Han, Mary E. Slaughter, Susan H. Godley, Bryan R. Garner

**Affiliations:** 1 RAND, 1776 Main Street, Santa Monica, CA 90407-2138 USA; 2 RAND, 4570 Fifth Ave., Suite 600, Pittsburgh, PA 15213 USA; 3 Chestnut Health Systems, 448 Wylie Dr, Normal, IL 61761 USA; 4 Research Triangle Institute, 3040 E. Cornwallis Rd. Research Triangle Park, Raleigh, NC 27675 USA

**Keywords:** Substance use treatment, Adolescent, Program sustainment

## Abstract

**Background:**

Few empirical studies longitudinally examine evidence-based practice (EBP) sustainment and the hypothesized factors that influence it. In an effort to address this gap, the current study examined sustainment of an EBP for adolescent substance use called the adolescent community reinforcement approach (A-CRA).

**Methods:**

A-CRA sustainment was assessed via information collected as part of key informant interviews and surveys with clinical staff from community-based treatment organizations that had received federal funding to implement A-CRA. Administrative data from the funding period on implementation was also used. Using discrete-time survival analysis, we regressed A-CRA sustainment on several factors theorized to influence EBP sustainment. Factors examined included outer setting, inner setting, implementation quality during the funding period, and intervention-related characteristics.

**Results:**

Overall, data from 83 % of the targeted sample of treatment organizations was collected. A-CRA sustainment was strongly related to the time since funding loss. Strong relationships were found between sustainment status and implementation quality during the funding period, agency focus, funding stability, and political support for the treatment along with staff perceptions of the treatment’s complexity and implementation difficulty.

**Conclusions:**

Consistent with the Consolidated Framework for Implementation Research, the current study found several factors related to the outer setting (e.g., funding stability), inner setting (e.g., agency focus), implementation quality during the funding period (e.g., staff trained, clients served), and characteristics of the intervention (e.g., implementation complexity) to be associated with EBP sustainment. Future research is warranted to examine the extent to which these relationships are stable over time. Efforts to ensure that adequate implementation occurs during the initial implementation period and that adequate funding, infrastructure, and staff support following the ending of initial support are critical to a program’s survival.

## Background

In order to improve health care quality, many government agencies and community organizations have invested significant resources to encourage evidence-based practice (EBP) implementation with the hope that adoption of EBPs will lead to improved health care outcomes. This is particularly relevant for the treatment of adolescent substance use disorders for several reasons. First, the quality of adolescent treatment services has been known to be highly variable [1, 2] with fewer than half of those receiving treatment achieving recovery a year following treatment [3]. Second, treatment for substance use disorders has been slow to be integrated into mainstream medical care settings and has heavily relied on community and peer-based approaches that lack a strong theoretical and empirical base. Moreover, developmentally appropriate treatments for adolescents are often not incorporated into practice settings [4, 5]. Providing EBPs may mitigate the future consequences for involved adolescents and help to reduce the public burden of caring for a chronic relapsing condition that is estimated to cost in excess of $600 billion a year [6–8].

In order to address this critical gap, the Substance Abuse and Mental Health Services Administration’s (SAMHSA) Center for Substance Abuse Treatment (CSAT) has offered discretionary grant funding to community-based treatment organizations in order to help facilitate the delivery of EBPs for adolescent substance use disorders. Among one of the largest investments to date has been support for an EBP called the adolescent community reinforcement approach (A-CRA). A-CRA is a behavioral intervention that seeks to replace environmental contingencies that encourage substance use with pro-social activities and behaviors that promote recovery. Three randomized controlled studies of A-CRA have yielded positive outcomes across alcohol use, mental health, and social domains [3, 9, 10]. Since 2006, SAMHSA has provided multi-year funding to 84 community-based treatment organizations located in over 27 states/districts/territories across the USA and has totaled more than 150 million dollars in funding. The discretionary grants that treatment organizations received were 3 years of implementation support, which included approximately $300,000 annually, 2.5 days of in-person A-CRA training followed by a standardized clinical and supervision certification process that incorporated technology-assisted performance feedback [11].

Despite this large investment to support EBP implementation, little is currently known about how effective these grant mechanisms are in supporting the sustainment of A-CRA following the SAMHSA funding period. That is, the long-term effectiveness of these investments in improving longer-term EBP implementation is unknown. To address this question, we looked to the burgeoning field of implementation science to inform our research hypotheses. We found a diversity in the definitions of practice sustainment and sustainability [12–18] and conceptual frameworks [12, 19–26] regarding implementation; however, across this literature, we found four main factors theorized to influence sustainment: (1) the broader community environment, external to the organization implementing the practice (i.e., “outer setting”), for example, policy and fiscal factors; (2) the level or extent of implementation (or implementation quality) during the funded and/or initial period; (3) factors within the organizational or “inner” setting, such as strategic planning, program evaluation, and program adaptation; and (4) elements of the practice itself, that is, the intervention characteristics and staff perceptions of the treatment. Although each of these factors may conceptually play a role in EBP sustainment, we posit that such factors as external funding and political factors may trump such factors as inner setting activities which include strategic planning, evaluation, and communication.

### Previous literature

There has been an increased attention to assessing EBP sustainment [27], and a recent literature review of over 125 studies found that the research on sustainability within the health care field is fragmented and underdeveloped [28]. In particular, few rigorous studies have been conducted using prospective methods. An exception, Tibbitts, Bumbarger, Kyler, and Perkins [29] examined the sustainment of crime and delinquency programming in school settings 1–3 years post-funding. The investigators found that leadership support, overall school support, adequate staffing, financial stability planning, and aligning the intervention with the setting (i.e., “fit”) were related to self-reported sustainment albeit with a small self-selected sample of programs. Since Wiltsey Stirman et al.’s review, Peterson et al. [30] examined prospectively the sustainability of five mental health evidence-based practices over an 8-year period. Leadership turnover appeared important, similar to previous studies [31]; however, implementation quality did not appear to predict long-term sustainment. This study was limited however to the study of internal (i.e., organizational or “inner setting” factors), and the authors argued for the need to incorporate external factors such as financial policies in future research. And most recently, Cooper, Bumbarger, and Moore [32] examined 2-year sustainment among 77 grantees who received seed money for youth delinquency and substance use prevention programs. Program staff were interviewed about whether the program was still operating and about community coalition support and readiness, program fit, staff characteristics, and sustainability planning utilizing a host of home-grown measures. They found that 69 % of the grantees reported program sustainment 2 years beyond initial seed funding, but of those, 60 % reported operating at a “lower level” than during the funding period. Predictors of sustainment included improved coalition functioning, greater outreach to community stakeholders, and sustainability planning. Program “fit” as defined by such factors as participant recruitment, engagement, knowledgeable well-trained program staff, and administrator support was also found to be related to sustainment. The study however was limited to studying sustainment at 2 years post seed funding among prevention programs in Pennsylvania.

In sum, limitations of the previous research include the lack of empirical, longitudinal studies and the use of small select samples. There is also little information about the sustainment of EBPs in relation to behavioral therapies for the treatment of substance use which is typically provided in community-based settings that are often low or under-resourced and experience high staff turnover [33]. Therefore, to help address limitations in past research, the current study sought to examine factors associated with the sustainment of A-CRA in order to understand the facilitators and barriers to its continued implementation following federal support for its delivery. This is important because the SAMHSA/CSAT continues to provide support to deliver A-CRA along with other behavioral treatments to address substance use. In this study, associations between theorized implementation factors and sustainment are examined using data collected from federally funded community-based adolescent substance abuse treatment organizations during the 3-year implementation period and from key organization staff following the 3-year funded period. Understanding the factors associated with EBP sustainment may assist in identifying and developing appropriate supports to ensure the large investments in program implementation lead to long-term benefits for the communities that the programs serve.

## Methods

### Study context

This project examines A-CRA sustainment among community-based adolescent substance use treatment programs that were awarded funding by the SAMHSA/CSAT between 2006 and 2010. During that period, there were four program cohorts funded by the SAMHSA/CSAT called the “Assertive Adolescent Family Treatment” initiative in 2006, 2007, 2009, and 2010 (e.g., [34]). For these initiatives, grantees were required to utilize A-CRA as the treatment approach. In addition, other SAMHSA funding opportunities were offered during this period including the “Juvenile Drug Court” and “Juvenile Drug Treatment Court,” the “Offender Reentry Project,” and the “Targeted Capacity Expansion” initiatives. For these initiatives, the community-based grantee was required to identify an EBP, and several of the funded organizations selected A-CRA and therefore were included in our study sample. At the time of data collection for this study (Fall 2013), the cohorts varied in the time since the loss of federal funding from approximately 48 months to 1 month.

### Sample

#### Organizations

The study sample was composed of nonprofit treatment providers located across the country representing 27 states. Using recent data available from the National Survey of Substance Abuse Treatment programs [35], 88 % of adolescent treatment is provided in outpatient settings, and 66 % of that treatment is delivered by nonprofit providers, similar to the proposed study sample. The funder also specified that applicants were required to demonstrate (a) program operation in the same geographical location(s) for at least 2 years prior to the proposed project period and (b) compliance with local and state/tribal licensing, accreditation, and certification requirements. These specifications indicated SAMHSA’s intent to build existing substance use treatment program capacity rather than to support new programs. In some cases, the grantee was a non-substance use treatment provider (e.g., school, court, community organization) that partnered with an existing substance use treatment provider in order to deliver the services.

#### Staff

Clinical supervisors and clinicians at the treatment organizations were recruited to participate. We aimed to enroll at least two individuals from each program that were familiar with A-CRA and had experience providing A-CRA services directly to youth and/or supervising staff providing A-CRA services.

### Data collection

#### Data sources

Primary data was collected from interviews and surveys with organization staff. Implementation data collected during the funding period was also used.

#### Data collection procedures

We used an administrative dataset that provided the research team with the contact information of the funded treatment organizations and the staff at the treatment organizations who were trained during the 3-year funding period to implement A-CRA. Our recruitment strategy employed multiple methods (i.e., mail, phone, and email) to introduce and remind potential participants about the study, consistent with effective tailored survey methods [36]. We first introduced the study via an email to clinical supervisors and clinical staff. We followed up the introductory email with phone calls to request and/or confirm an interview time. Once an interview was completed, participants were sent an email with instructions on how to access the online survey. Final attempts to contact participants for those that did not respond by email or phone were sent by FedEx.

### Measures

#### A-CRA sustainment

For the analyses reported in this paper, we used staff reports of whether the organization currently utilized the A-CRA treatment or not at the time of the interview. For treatment organizations that reported no longer using A-CRA, we asked when they stopped using it to document the length of time following the federal funding period that the organization utilized A-CRA. From administrative data regarding implementation, we had information about when the organization first received federal funding to deliver A-CRA (i.e., grant start date) and when the organization stopped receiving federal funding (i.e., grant end date) so that we could calculate the time since the federal funding grant end date that the A-CRA treatment was sustained. The funding period was typically 3 years, but some organizations were granted no-cost extensions for 6 to 12 months. We utilized the number of months since grant end date to characterize A-CRA sustainment.

#### Implementation characteristics

Consistent with several theories of implementation [19, 26], we selected measures that characterized the four main factors theorized to be related to sustainment: inner and outer setting characteristics, implementation factors, and intervention characteristics. As few empirical studies exist in the area of program sustainment, there is an increasing literature on developing valid assessment tools related to the hypothesized constructs. As noted in more detail in the following sections, we attempted to assess these four main constructs using measures that have been recently developed for this purpose (e.g., Program Sustainability Assessment Tool (PSAT)) or have been used in previous studies related to program implementation (e.g., the Steckler and O’Loughlin tools) while ensuring we capture aspects that were particularly relevant to the study context (i.e., adolescent substance use treatment). Data for these measures were collected from staff participants as part of an online survey following a phone interview where they discussed whether their organization was still implementing A-CRA treatment with adolescents.

#### Setting characteristics

Both Greenhalgh et al. (2004) [21] and Damschroder et al.’s (2009) [19] theories specify that structural characteristics, such as the size and architecture of an organization may influence implementation. We operationalized this by examining the comprehensiveness of the organizations, by evaluating a count of services offered at the organization using a survey question from the National Survey of Substance Abuse Treatment Services [37]. The range on this variable was from 0–17.

“Innovation-fit” has also been nominated as an important factor in implementation [19, 21] and sustainment theories [26]. We operationalized this construct at the organizational level by asking what the primary focus of the organization at the location in question was with the following response options: substance use treatment services, mental health services, a mix of mental health and substance use services (neither is primary), general health care, and other. A binary variable was used that indicated whether staff reported that substance use treatment was the primary service (coded “1”) as compared to all other options (coded “0”).

To meet the goals of developing measurement tools related to program sustainability, a team at Washington University recently conducted a literature review, expert panel, and concept mapping to develop the Program Sustainability Assessment Tool [38, 39] which consists of eight subscales: communication (e.g., “The program has communication strategies to secure and maintain public support”), funding stability (e.g., “The program has a combination of stable and flexible funding”), organizational capacity (e.g., “The program is well integrated into the operations of the organization”), partnerships (e.g., “Diverse community organizations are invested in the success of the program”), political support (e.g., “Political champions advocate for the program”), program adaptation (e.g., “The program adapts strategies as needed”), program evaluation (e.g., “Evaluation results inform program planning and implementation”), and strategic planning (e.g., “The program plans for future resource needs”). Each subscale consisted of five items and the alphas among our samples ranged from 0.84–0.95. Responses ranged from “to little or no extent” (scored as a “1”) to “to a great extent” (scored as a “7”), we summed responses (for a range of 5–35) and calculated mean values for each scale. This assessment tool encompasses both outer and inner setting characteristics.

#### Implementation characteristics

The extent to which an organization has consistently implemented the intervention during the funding period is likely to influence how well it can be sustained post the initial support period [26, 40]. In order to examine this, we utilized data collected during the funding period that included (1) the number of adolescents that received A-CRA during the funding period, (2) the number of clinical supervisors certified in A-CRA and still employed at each organization at grant end, and (3) the number of clinicians certified in A-CRA at each organization and still employed at the end of the grant period. More specifically, the number of participating adolescents was based on the number of adolescents who completed a baseline interview. The number of staff who completed the A-CRA clinical supervisor and clinician certification process and were still employed at the end of the grant period at each organization was drawn from data collected by the technical assistance group that provided the A-CRA training and monitored implementation for the SAMHSA/CSAT. We intentionally selected assessments of the number certified staff employed at grant end, rather than the number of staff trained during the funding period or staff turnover, to more accurately account for the organizational resources at grant end that may influence sustainment.

#### Intervention characteristics

Several theories suggest that perceptions of a particular innovation in terms of its ease of use and benefit over alternative options will influence its adoption, use, and presumably longer-term sustainment [19–21, 26]. We included assessments of staff perceptions of A-CRA’s complexity (e.g., “A-CRA is hard for therapists to understand”; using a scale of five items) and relative advantage (e.g., “A-CRA is more effective in reducing substance use by clients than our current treatment practices”; using a scale of four items) using items from Steckler et al. [41] (alphas = 0.88 and 0.83, respectively). We included staff perceptions of implementation difficulty (e.g., “Recruiting staff/participants for A-CRA is difficult”) and perceived success (e.g., “A-CRA had an impact on participants”) using five-item scales developed from O’Loughlin et al. [16] (alphas = 0.57 and 0.91, respectively). All of these survey items had response options on a five-point scale ranging from “strongly disagree” (scored as a “1”) to “strongly agree” (scored as a “5”) for a summed score range of 4–20 (for the relative advantage scale) or 5–25 (for the complexity, difficulty, and success scales).

#### Analytic plan

We conducted a set of discrete-time survival analyses. The self-reported termination of A-CRA was defined as the event occurrence in the survival analysis. The time-to-event was the number of months between the end of federal funding and the time an organization stopped implementing A-CRA. The time-to-event was right censored by our interview time, and the censoring was independent of the time-to-event. We first fitted a Kaplan-Meier survival curve (i.e., the probability that an organization sustains A-CRA longer than a given length of time). Next, we examined the marginal proportional hazards (i.e., the ratio in hazards between two levels of a binary factor or a unit change of a continuous predictor, where the hazard is the conditional probability that an organization ceases to sustain A-CRA at the end of a month given it sustains A-CRA at the beginning of the month) for a list of hypothesized factors. Organization-level values for the hypothesized outer, inner setting, implementation quality, and intervention characteristics were computed by taking the mean (for scale variables) or mode value across the participant responses at an organization. Missing data at the organizational-level was imputed by mean imputation conditioned on the sustainment statuses. The discrete-time marginal proportional hazard was estimated by a logistic regression following the standard approach to recode the time-to-event data to binary outcomes [42, 43]. To account for multiple comparisons, we apply the step-up methods to adjust *p* values to control the false discovery rate at the .05 and .10 levels [42].

## Results

### Sample characteristics

Of the 84 treatment organizations that were funded between 2006 and 2010, 82 programs had lost funding at the time of data collection. Staff from 68 organizations participated in the data collection for an 83 % response rate. The range in months of time since funding loss ranged from 1–48 months (see Table 1). Response rates were not related to time since funding loss as we found no significant relation between funding year and participation rate (*χ*^2^ (4, *N* = 68) = 3.53, *p* = 0.53). We also examined whether there were differences among the sites that responded and those that did not respond on the implementation variables (i.e., the number of youth treatment, the number of certified staff employed at grant end) and did not find any significant associations between sites responding and implementation.

**Table 1:** Estimated survival probability by time since funding loss for participating organizations

Time since funding loss (months)	No. of sites at risk	No. of sites at risk that stopped delivering A-CRA	Estimated survival probability (%)
<1	68	9	86.8
1–12	42	11	64.0
13–24	10	0	64.0
25–36	6	2	42.7
>36	1	0	42.7

### Probability of A-CRA sustainment

**Fig. 1:**
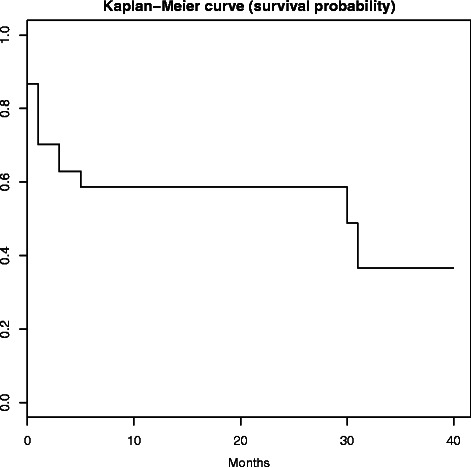
The estimated Kaplan-Meier survival probability that an organization sustains A-CRA longer than a given time

Based on the estimated Kaplan-Meier survival curve, the survival probability (i.e., the probability that an organization sustains A-CRA longer than a given time) was 86.8 % (95 % CI 79.1 %, 95.2 %) at the beginning of the first month after the CSAT grant funding ended and 58.7 % (95 % CI 44.8 %, 76.7 %) at the end of the first year after grant funding ended (see Fig 1). The estimated survival probability remained flat until the 32nd month, where it dropped to 36.7 % (95 % CI 17.8 %, 75.4 %). The 95 % confidence intervals at the 32nd month were wide because very few organizations were between 2 and 4 years after funding ended at the time of the current study.

### Factors associated with A-CRA sustainment

Mean values or percentages endorsing the hypothesized factors by A-CRA sustainment status are presented in Table 2. The estimated proportional hazards (PH), i.e., the ratio in hazards between two levels of a binary factor or a unit change of a continuous predictor between the mean imputation and the multiple imputation methods, were very similar. Table 3 presents the estimated proportional hazards based on the mean imputation, where a negative PH indicated a lower hazard or higher probability to sustain A-CRA when the factor value was increased, and vice versa. Standard errors, 95 % Wald confidence levels, and chi-square statistics for each model are also presented in Table 3.

**Table 2:** Descriptive statistics on the hypothesized factors by program sustainment status

Characteristics	Non-sustainers	Sustainers
	(*n* = 22)	(*n* = 46)
	M (SD)/%	M (SD)/%
Setting		
Organizational focus (% substance use)	22.73	67.39
No. of services offered	11.91 (3.04)	12.24 (2.5)
Communications	23.95 (4.47)	22.95 (7.61)
Funding stability	13.24 (3.67)	19.12 (6.51)
Organizational capacity	26.37 (6.06)	24.73 (6.46)
Partnerships	19.88 (6.84)	21.41 (6.73)
Political support	21.87 (6.06)	23.63 (5.97)
Program adaptation	28.30 (3.81)	26.27 (4.95)
Program evaluation	27.61 (4.23)	26.45 (5.19)
Strategic planning	20.37 (5.03)	22.03 (6.86)
Implementation		
No. of clinicians certified/employed at grant end	1.36 (1.26)	2.44 (1.51)
No. of supervisors certified/employed at grant end	0.91 (0.87)	0.91 (0.72)
No. of youth served during grant period	91.82 (48.28)	128.8 (100.9)
Intervention		
Complexity	7.77 (2.41)	5.78 (1.95)
Implementation difficulty	16.27 (3.12)	14.85 (2.84)
Perceived success	20.83 (3.90)	20.51 (2.90)
Relative advantage	15.86 (3.65)	16.33 (1.93)

**Table 3:** Models estimating the marginal proportional hazards of stopping the sustainment of A-CRA

Characteristics	Estimate	SE	Lower	Upper	Chi-square	*p* value
			Wald CL	Wald CL		
Setting						
Org. focus	−1.90	0.53	−2.93	−0.86	12.95	<0.001*
No. of services	−0.06	0.07	−0.21	0.09	0.57	0.45
Communications	0.02	0.03	−0.04	0.08	0.42	0.52
Funding stability	−0.20	0.05	−0.31	−0.09	13.64	<0.001*
Org. capacity	0.04	0.04	−0.03	0.12	1.22	0.27
Partnerships	−0.01	0.04	−0.08	0.07	0.03	0.87
Political support	−0.09	0.04	−0.16	−0.01	4.92	0.03**
Prog. adaptation	0.08	0.05	−0.03	0.18	2.13	0.14
Prog. evaluation	0.05	0.05	−0.04	0.14	1.06	0.30
Strategic planning	−0.01	0.03	−0.08	0.06	0.06	0.81
Implementation						
No. of clinicians	−0.88	0.24	−1.36	−0.41	13.16	<0.001*
No. of supervisors	−0.32	0.32	−0.94	0.31	1.00	0.32
No. of youth	−0.01	0.01	−0.02	−0.01	4.93	<0.03**
Intervention						
Complexity	0.41	0.11	0.20	0.62	14.27	<0.001*
Imp. difficulty	0.21	0.09	0.04	0.38	5.60	0.02**
Perc. success	−0.03	0.08	−0.18	0.12	0.18	0.67
Rel. advantage	−0.13	0.08	−0.29	0.03	2.48	0.11

*SE* standard errors, *CL* confidence levels

*Statistical significance at false discovery rate of 0.05

**False discover rate of 0.10

When the false discovery rate was controlled at the level of .05, factors significantly associated with A-CRA sustainment included an organization whose focus was substance use (rather than mental health, combination mental health, and substance use or something else) (PH = −1.90), the number of clinicians with basic A-CRA certifications still employed at the organization at the end of the federal funding period (PH = −.88), staff perceptions of A-CRA’s complexity (PH = 0.41), and A-CRA funding stability as assessed by the PSAT scale (PH = −0.20). When the false discovery rate was controlled at the .10 level, three additional factors were significantly related to sustainment: staff’s perception of implementation difficulty (PH = 0.21), political support as assessed by the PSAT scale (PH = −0.09), and the number of adolescents treated with A-CRA during the funding period (PH = −0.01).

## Discussion

Using data collected from key clinical staff, we found that the sustainment of an evidence-based adolescent treatment protocol was likely to eventually be discontinued by a majority of programs within 3–4 years after the initial federal funding ended. More specifically, about two thirds of the organizations reported A-CRA sustainment at 12–24 months following the end of funding. However, fewer than half the programs continued to sustain A-CRA 36–48 months after funding ended. These findings are comparable to those reported by Cooper et al. [32] regarding seed funded prevention programs at 2 years post-funding (69 %) and by Scheirer who conducted a review of community coalition health-related program studies [44] (60 %). We are not aware of any comparable studies that focus on the sustainment of substance use treatment or more specifically, adolescent treatment programs which may be more likely to sustain themselves using a different organizational structure and funding mix than programs operated by community coalitions or prevention programming that is often embedded in schools settings.

In terms of factors that appear related to A-CRA sustainment, we found that setting-, implementation-, and intervention-related characteristics were important. The type of organization that was funded, the level of implementation during the funding period, funding stability, political support for the program, and staff perceptions of A-CRA’s complexity and implementation difficulty were related to A-CRA sustainment. These findings are consistent with previous theories that suggest that existing infrastructure, such as the intervention being consistent with the overall mission of the organization, is key to adoption, implementation and sustainment [45, 20, 19]. More specifically, one of the most compelling findings was staff that primarily described their organization as substance use focused, rather than mental health, general health care, or some other focused were more likely to report sustaining A-CRA. Although A-CRA has been demonstrated to be effective among adolescents with co-occurring mental health disorders and the majority of adolescents in these programs are referred from criminal justice settings [46–48], it appears that substance use treatment-oriented organizations are better equipped to continue delivering an adolescent substance use treatment program than organizations with a mental health or other type of focus. The findings appear to also be consistent with the Tibbitts et al. [29] who found that organizational (in this case, school) support and program “fit” were related to 1- to 3-year program sustainment and several previous studies that emphasize that funding stability is the primary element to program sustainment [44]. For example, following the federal initiatives that supported the participating organizations to deliver A-CRA, federal support for adolescent treatment has been allocated to single state agencies for substance use that can then grant funds to local community-based organizations that have existing capacity to deliver substance use treatment (e.g., SAMHSA CSAT’s Cooperative Agreements for State Adolescent and Transitional Aged Youth Treatment Enhancement and Dissemination [49]). Therefore, organizations with a demonstrated track record for delivering substance use treatment may be better positioned to receive future funding to support adolescent treatment.

It is also somewhat surprising that a host of factors typically associated with program adoption and implementation, such as staff perceptions of organizational capacity, program planning, communication, and evaluation activities were not found to be significantly associated with sustainment. Although there often efforts by funders to build the evaluation capacity of their grantees and assist them with strategic planning and communication about their program as well as help form partnerships among local program stakeholders, it appears that existing in a stable funding environment, regardless of evaluation and planning efforts, is critical to a program’s long-term survival.

The results may appear somewhat inconsistent with the previous research. For example, we found that the level of implementation at the end of the funding period (as assessed by the number of staff certified and employed at grant end and the number of youth served during the grant period) was associated with sustainment, whereas Peterson et al. [30] reported that few indicators of implementation quality were related to sustainment. It is important to note the Peterson et al. [30] study used assessments that represented longer time points (i.e., 2 years apart) and included implementation measures that were different from ours (e.g., staff turnover). We selected a more refined capacity measure than simply staff turnover, as we took into account the organization’s capacity at the end of the funding period (i.e., number of staff certified to deliver the treatment), which may have been more closely associated to sustainment than attrition rates. Given the lack of empirical studies on this topic, it is not unusual that the few existing studies use different time periods and assessment tools. More research is needed to help replicate these findings.

The study findings also diverged from those found by Cooper et al. [32] in that community support, and external partnerships were not found as an important factor associated with program sustainment. This inconsistency may be due to the differences between studied programs; Cooper et al. [32] focused on newly funded prevention programs which may require more community level support than established treatment programs as prevention programs are often built by community coalitions rather than being primarily operated within one particular organization, like found with many substance treatment programs.

In sum, we found that organizational focus, funding stability, political support, implementation quality during the funding period, and perceptions of the intervention in terms of complexity and implementation difficulty by key staff are important factors associated with program sustainment. These factors appear to be more critical to sustainment than many of the organizational factors often hypothesized as important to implementation (and thus sustainment) including staff perceptions of other organizational factors, such as program planning, communication, and evaluation activities.

We address several limitations to previous research on health care program sustainment. For example, to advance what is known about program sustainability, researchers have argued that it is necessary to study sustainability over several years rather than just a single point in time [28]. In this study, some organizations that we queried were just ending their initial federal funding support and others that had lost funding up to 4 years ago. Moreover, few past studies have used conceptual frameworks to inform their work. This study was developed taking into account several existing conceptual approaches to program implementation and sustainment, and therefore, we assessed factors both external to and internal to the organization along with intervention-specific components to sustainment. Also, few studies have employed longitudinal, prospective methods. Given the extensive data collected during the implementation period, we were able to examine several variables prospectively and demonstrate that such factors as the number of adolescents exposed to the treatment and the number of clinical supervisors and clinicians certified to deliver the treatment and still employed at the end of the funding period were both predictive of later A-CRA sustainment. These implementation factors appear especially important to predicting sustainment and represent more “objective” characteristics than staff perceptions and attitudes collected in the post-funding period. In sum, this study addresses many important gaps in previous research.

It is important to note that this study represents the first of 3 years of longitudinal data that are being collected. In the future, we plan to report on whether these findings are stable using a larger sample of organizations that have matured several years beyond the federal funding period. In addition, we will incorporate both qualitative and quantitative data to better explain the relationships between theorized factors and A-CRA sustainment. For example, thus far, we have learned from staff that competing funding requirements, that is, situations where local, state, or federal funding are tied to a different EBP sometimes shift the organization to a different treatment regime even though there is an organizational support for the treatment, and staff perceive it as an effective approach [50].

Some limitations to this study are important to acknowledge. First, this study relied on self-reported sustainment from key clinical staff charged with treatment delivery. We focused on reports from clinical staff, as they are most likely to be aware of the treatments offered and what adolescents received, as compared to administrative staff. However, self-reports may be subject to bias and may not represent the extent to which a treatment is delivered with fidelity. Our future work is to better explicate A-CRA sustainment through the assessment of core elements of the treatment as specified by the treatment developer (e.g., delivery of an adequate number of sessions and ongoing clinical supervision that is aligned with the treatment developer’s approach used during the funded implementation period). A second limitation to this study is the relatively small sample. We targeted the entire population of organizations that were funded and achieved over a 80 % response rate; however, to study the multitude of hypothesized factors and the potential interaction effects, a larger sample would be required; this is a commonly noted challenge in implementation research where the main analysis is often conducted at the organizational rather than individual level.

### Conclusions

Only one in 20 youth in need of substance use treatment receives it [35]. Of those in treatment, less than half are positively discharged from treatment [51], suggesting the need for the practice of effective treatments. Despite the fact that the A-CRA has demonstrated effectiveness, and that most treatment providers sustained delivery of it initially after funding ended, we found that longer-term sustainment was challenging. Successful implementation during initial funding period appeared an important factor to longer-term sustainment along with the organization’s focus, funding stability, political support for the treatment, and positive perceptions about the treatment by clinical staff. As government and other entities consider support for the implementation of EBPs, it is important for them to consider what types of settings, infrastructures, and organizational factors should be present during the selection process to ensure their investment is well spent.

## Abbreviations

A-CRA: adolescent community reinforcement approach
CI: confidence interval
CL: confidence level
CSAT: Center for Substance Abuse Treatment
EBP: evidence-based practice
M: mean
N-SSATS: National Survey of Substance Abuse Treatment Services
PH: proportional hazard
SAMHSA: Substance Abuse and Mental Health Services Administration
SD: standard deviation
SE: standard error
